# Supplementary material for: Estimating incidence of infection from diverse data sources: Zika virus in Puerto Rico, 2016
Source: PLoS Comput Biol. 2021 Mar 30;17(3):e1008812. doi: 10.1371/journal.pcbi.1008812 (PMC8034731; doi:10.1371/journal.pcbi.1008812)
Supplement: S1 Text — This supplement contains additional parameter descriptions and additional tables and figures. (DOCX) [file pcbi.1008812.s001.docx]

**Supplemental Information**

*Priors for blood bank model parameters*

We assumed the proportion of asymptomatic infections, $p_{A}$, came from a Beta distribution: $p_{A} \sim\mathrm{Beta}(0.68, 0.02) ADDIN EN.CITE ADDIN EN.CITE.DATA Error! Bookmark not defined.$[1-3]; the duration of viremia came from a Weibull distribution: $V\sim\mathrm{Weibull}\left( 9.97, 15.54 \right)$[3] and the incubation period, $D$, came from a Normal distribution: $D\sim Normal\left( 6, 0.23 \right) ADDIN EN.CITE ADDIN EN.CITE.DATA Error! Bookmark not defined.$ [3,4].

**Table A. Prior distributions of model parameters using informative, naïve, and increased variances**

| **Parameter** | **Informative** | **Increased variance** | **Naïve** |
| --- | --- | --- | --- |
| Transmission rate ($\beta_{t}$)  | $\mathrm{Normal}_{\left( 0,\infty\right)}(2, 1)$ | $\mathrm{Normal}_{\left( 0,\infty\right)}(2, 2)$ | $\mathrm{Normal}_{\left( 0,\infty\right)}(0, 10)$ |
| Baseline GBS risk $(p_{G0})$  | $\mathrm{Beta}(23, 8.9 x {10}^{7})$ | $\mathrm{Normal}_{\left( 0,\infty\right)}(2.5 x {10}^{-7}, 1 x {10}^{-7})$ | $\mathrm{Normal}_{\left( 0,\infty\right)}\left( 0, 100 \right)$ |
| Probability of Suspected GBS given ZIKV infection $(p_{G\vert Z})$  | $\mathrm{Beta}(5.9, 2.3 x {10}^{4}$)  | $\mathrm{Normal}_{\left( 0,\infty\right)}(2.6 x {10}^{-4}, 2 x {10}^{-4})$ | $\mathrm{Normal}_{\left( 0,\infty\right)}(0, 10)$ |
| Probability of a suspected case being reported ($p_{S\vert Z})$ | $\mathrm{Beta}\left( 3.3, 27 \right)$ | $\mathrm{Normal}_{\left( 0,\infty\right)}($0.11, 0.12) | $\mathrm{Normal}_{\left( 0,\infty\right)}(0.5, 0.5)$ |
| Relative incidence of ZIKV in the general population compared to positivity in blood donor ($f_{BB})$ | $\mathrm{Gamma}(6.7, 7.6)$ | $\mathrm{Normal}_{\left( 0,\infty\right)}(0.88, 0.7)$ | $\mathrm{Normal}_{\left( 0,\infty\right)}(0, 10)$ |

Abbreviations: GBS, Guillain-Barré Syndrome; ZIKV, Zika virus

*
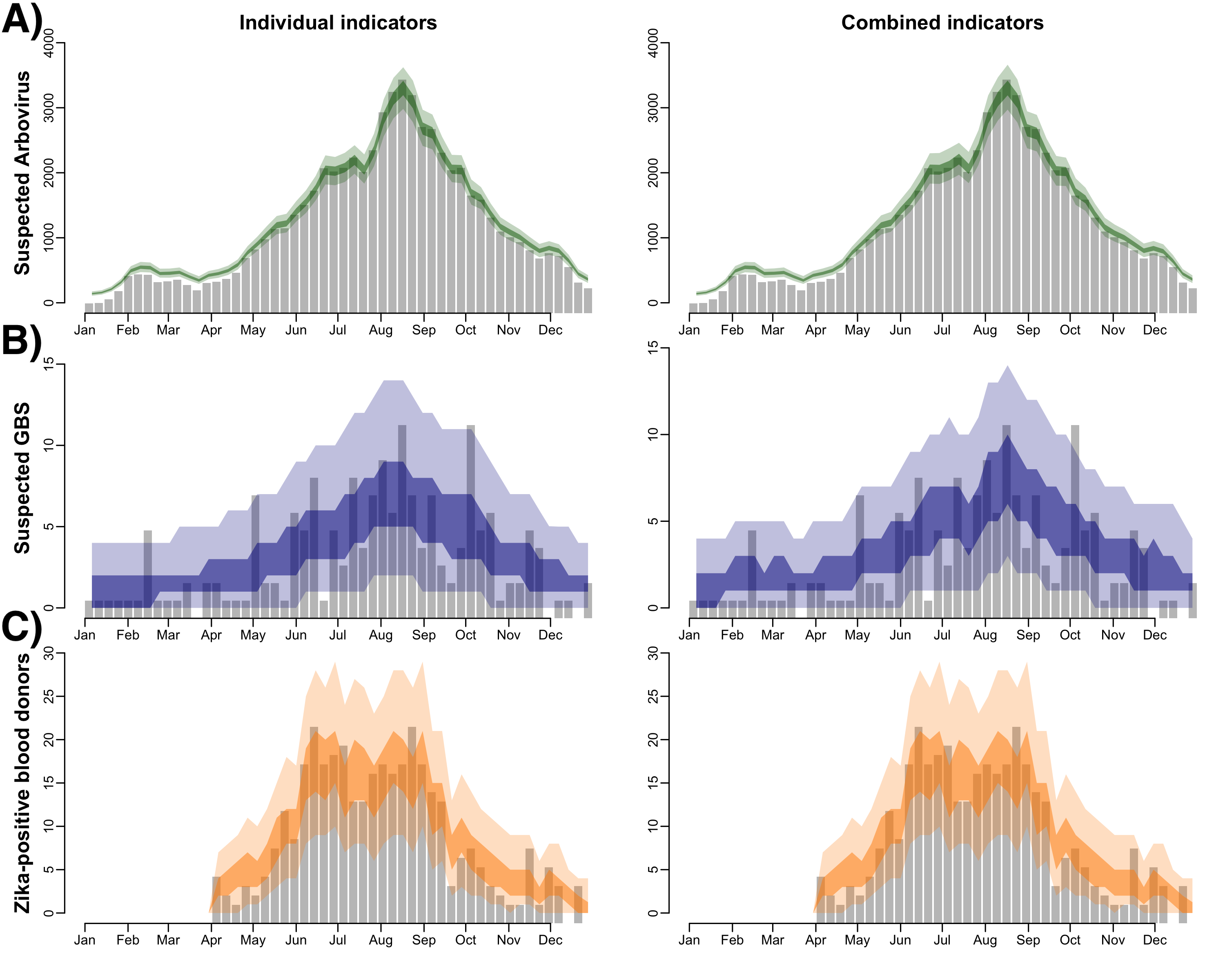
*

**Fig A. Reported suspected arbovirus cases, suspected ZIKV-associated Guillain-Barré Syndrome (GBS) cases, ZIKV-positive blood donors (grey bars) and estimated fits (shaded bounds) from informed individual indicator (left) and combined models (right column) during the 2016 outbreak in Puerto Rico**. A) Number of suspected arbovirus cases reported (green). B) Number of suspected ZIKV-associated GBS cases reported. C) Number of ZIKV-positive blood donors identified from blood donor screening. Dark bounds refer to the 50% range (interquartile range) of the credible interval (CrI) and lighter bounds refer to the 95% CrI.


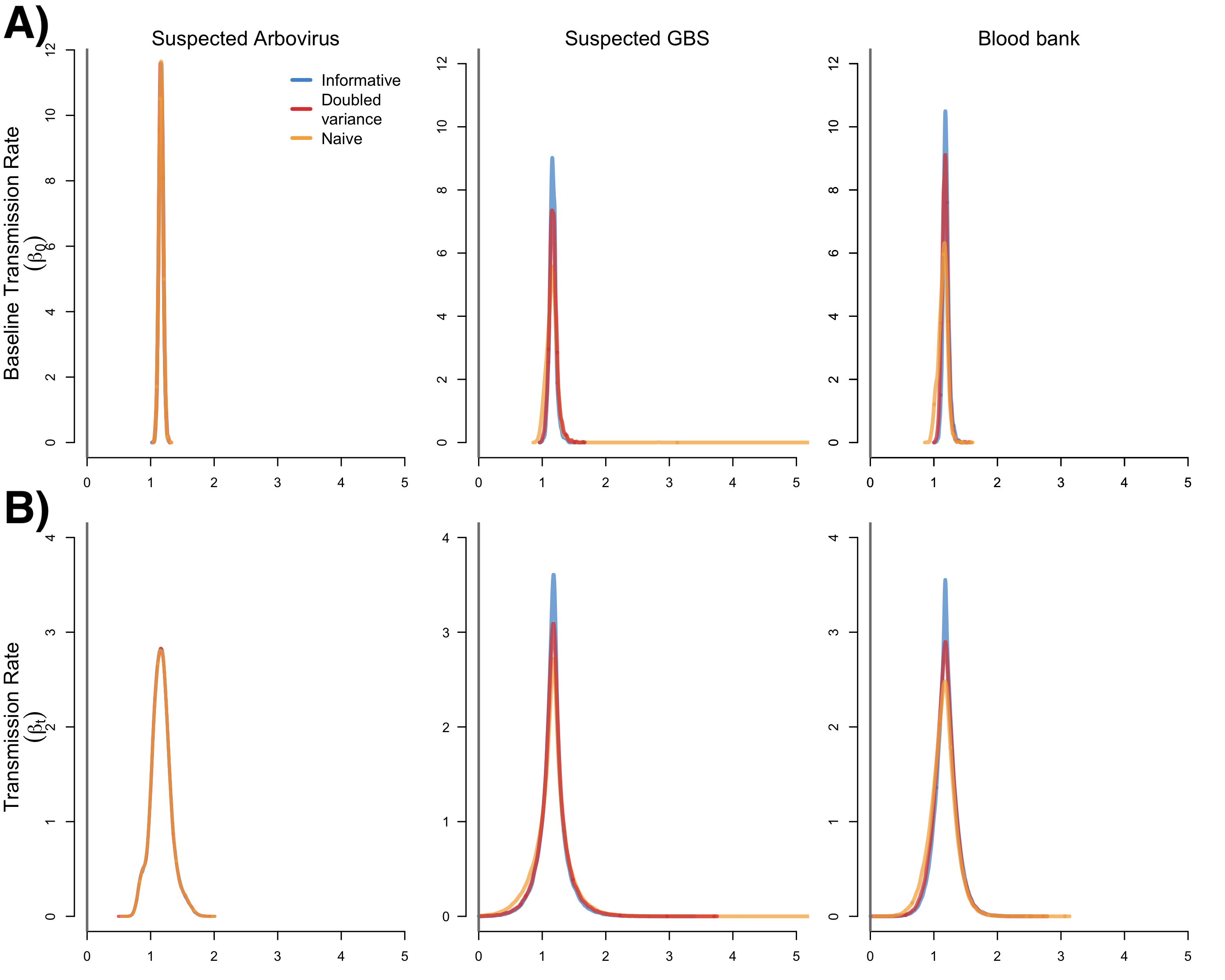


**Fig B. Posterior baseline and transmission parameters from the individual indicator models.** A) Posterior distributions of the baseline transmission parameter ($\beta_{0}$) from the Suspected Arbovirus, the Suspected GBS model, and the Blood bank data individual indicator models. B) Posterior distributions of transmission parameter ($\beta_{t}$) from the Suspected Arbovirus, the Suspected GBS model, and the Blood bank data individual indicator models. Color lines refer to assess variance assumptions of prior distributions in sensitivity analyses.

**
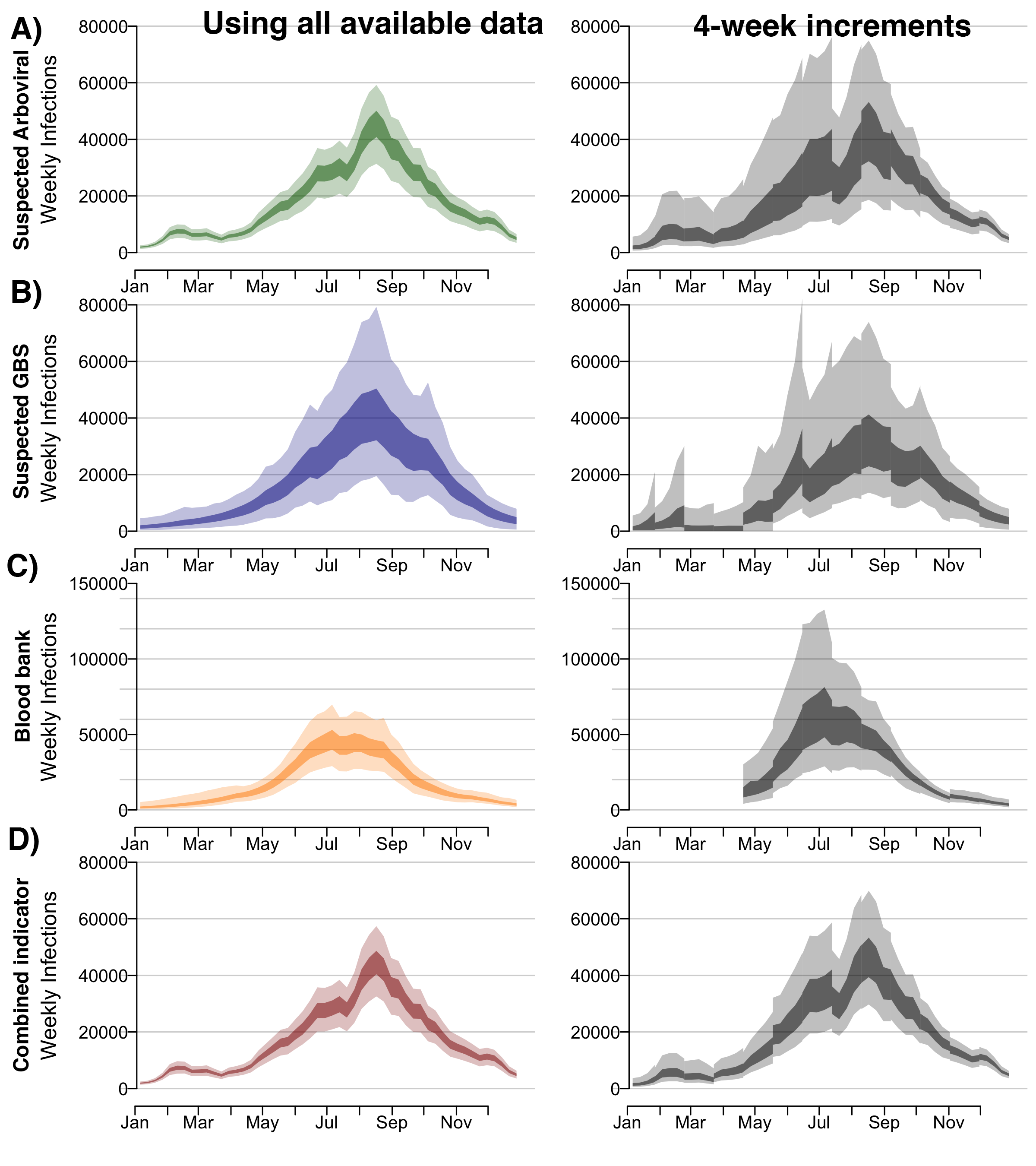
**

**Fig C. Estimated weekly Zika infections using all data (left) from the end of January 2016 to the end of December 2016) and estimated weekly Zika infections using increasing 4-week increments of data (right ) using A) Suspected arboviral indicator model, B) suspected GBS indicator model, C) Blood bank indicator model and D) a combined model using all three indicators.** Models using all available data estimated weekly infections over the course of the outbreak from January to the end of December. The four-week incremental approach used increasing amounts of data in four-week increments so that the models used the first four weeks of data available (i.e., data available in January), and increased the data used in the model by four-weeks, so that the second forecast used all available data for the first 8weeks, (i.e., data available in January and February), and so on. Because blood bank screenings did not identify cases until May, the four-week incremental model used to estimate weekly infections from blood bank data began forecasts in May. Dark bounds refer to the 50% range (interquartile range) and lighter bounds refer to the 95% credible interval (CrI).


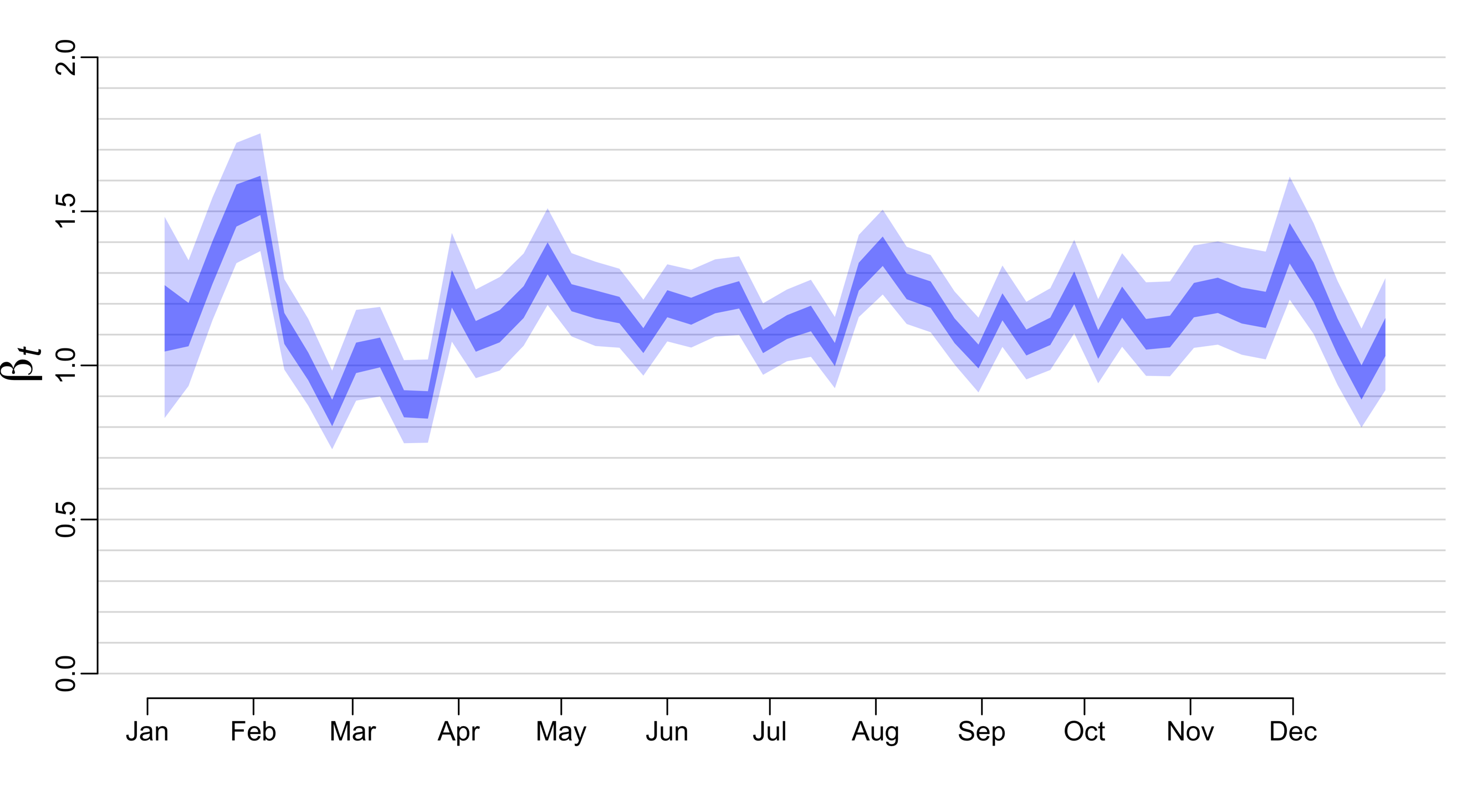


**Fig D. Weekly posterior estimates of the transmission parameter (**$\boldsymbol{\beta}_{\boldsymbol{t}}$**) from January 2016 to December 2016 using the informed combined indicator model.** The combined model parameters used informed priors. Dark bounds refer to the 50% range (interquartile range) of the credible interval (CrI) and lighter bounds refer to the 95% CrI.

References

1. Musso D, Nhan T, Robin E, et al. Potential for Zika virus transmission through blood transfusion demonstrated during an outbreak in French Polynesia, November 2013 to February 2014. Euro Surveill **2014**; 19.

2. Duffy MR, Chen TH, Hancock WT, et al. Zika virus outbreak on Yap Island, Federated States of Micronesia. N Engl J Med **2009**; 360:2536-43.

3. Chevalier MS, Biggerstaff BJ, Basavaraju SV, et al. Use of Blood Donor Screening Data to Estimate Zika Virus Incidence, Puerto Rico, April-August 2016. Emerg Infect Dis **2017**; 23:790-5.

4. Lessler J, Chaisson LH, Kucirka LM, et al. Assessing the global threat from Zika virus. Science **2016**; 353:aaf8160.
